# Vascular Stress Markers Following Inhalation of Heated Tobacco Products: A Study on Extracellular Vesicles

**DOI:** 10.1007/s12012-024-09934-6

**Published:** 2024-10-29

**Authors:** Lukasz Antoniewicz, Georgy Melnikov, Gustaf Lyytinen, Anders Blomberg, Jenny A. Bosson, Linnea Hedman, Fariborz Mobarrez, Magnus Lundbäck

**Affiliations:** 1 Department of Medicine II, Division of Pulmonology, Medical University of Vienna, 1090 Vienna, Austria; 2 Department of Clinical Sciences, Division of Cardiovascular Medicine, Danderyd University Hospital, Karolinska Institutet, Stockholm, Sweden; 3 Department of Public Health and Clinical Medicine, Umeå University, Umeå, Sweden; 4 Department of Public Health and Clinical Medicine, Section of Sustainable Health, Umeå University, The OLIN Unit, Umeå, Sweden; 5 Department of Medical Sciences, Uppsala University, Uppsala, Sweden

**Keywords:** Nicotine, Heated tobacco products, Heat not burn products, Endothelial function, Extracellular vesicles, Microvesicles

## Abstract

The advent of heated tobacco products (HTPs) has introduced new variables in the study of nicotine delivery systems and their health implications. Amidst concerns over cardiovascular effects, this study aims to elucidate the acute impact of HTP inhalation on extracellular vesicles (EV) levels in young, healthy individuals. In this controlled, acute exposure study, 23 young, healthy volunteers were subjected to HTP inhalation. EV levels of endothelial and platelet origin were quantified through flow cytometry before and after exposure. Data analysis was performed using multiple measures ANOVA to assess changes in EV concentrations. Our findings reveal a significant increase in EVs of endothelial and platelet origin following short-term HTP inhalation with nicotine. Notably, no significant change was observed in leukocyte- and neutrophil-derived EVs. This increase in EVs suggests acute vascular stress, with peak levels observed 4 h post-exposure. The rise in endothelial and platelet-derived EVs aligns with documented responses to acute vascular injury, paralleling the effects seen with traditional cigarette and e-cigarette use. Despite HTPs being marketed as safer alternatives, our results indicate that nicotine-containing HTPs may still pose significant vascular risks. These findings contribute to the growing body of evidence cautioning against the perceived safety of HTPs and reinforce the importance of regulatory oversight and public health initiatives targeting nicotine delivery technologies. **Trial Registrations:** ClinicalTrials.gov ID: NCT04824495, registered 2021–01-07.

**Supplementary Information:**

The online version contains supplementary material available at 10.1007/s12012-024-09934-6.

## Introduction

The World Health Organization (WHO) has identified cigarette smoking as a principal factor in premature mortality worldwide, responsible for an estimated 8 million deaths annually [1]. Amid innovations in nicotine delivery systems, including electronic cigarettes and nicotine pouches, heated tobacco products (HTPs) have gained prominence as contemporary alternatives. Launched by all major tobacco companies and propelled by vigorous marketing, HTP sales have grown substantially in the European Union [2].

HTPs operate by heating a prefabricated tobacco pod—commonly known as a heatstick or HEET—composed of tobacco sheets, water, guar gum, and cellulose fibers. Inserted into a battery-powered heating device, these pods are heated to around 350 °C to generate an aerosol inhaled by the user. Unlike traditional cigarettes, which combust at temperatures above 850 °C, HTPs reportedly employ a pyrolysis process, theoretically reducing the levels of toxic substances in the aerosol [3]. However, the notion that HTPs avoid combustion entirely is contested, with some studies indicating partial combustion [4, 5].

Limited data are available on the health impacts of HTP use, both when it comes to short and long term. Although HTP aerosols contain 90–95% fewer toxicants than conventional cigarette smoke [5–7], the presence of harmful chemicals persists. Notably, the nicotine yield of an IQOS heatstick approximates 70–80% of a standard cigarette’s nicotine content, with potential for indoor air pollution and secondhand exposure [6, 7].

Recent research underscores HTPs’ adverse effects on vascular and pulmonary health, including oxidative stress and lung injury in mice following HTP exposure and endothelial dysfunction in rats mirroring the impact of conventional cigarettes [8–10]. The long-term consequences of HTP usage, due to their relative novelty, remain uncertain.

Over recent decades, the significance of extracellular vesicles (EVs), including microvesicles —micrometer-sized vesicles shed from the cell membrane involved in cell–to–cell communication—has become increasingly recognized. Contrary to being mere cellular debris, EVs facilitate intricate signaling mechanisms, incorporating into recipient cells to exert biological effects [11]. Previous research has indicated the formation of endothelial and platelet-derived EVs in response to vascular stressors, such as myocardial infarction or stroke [12–14]. While the precise function of EVs in vascular stress remains to be fully elucidated, they are believed to play crucial roles in maintaining vascular homeostasis, possessing pro-thrombogenic and fibrinolytic properties as well as initiating repair mechanisms [15].

Our prior studies have documented significant increases in endothelial and platelet-derived EVs following cigarette and e-cigarette inhalation in young, healthy individuals, suggesting early signs of vascular stress [16, 17]. This study aims to extend these findings by investigating the effects of HTP inhalation on EV levels, alongside observed increases in arterial stiffness and thrombus formation, to elucidate the impact of HTPs on vascular health [18].

## Materials and Methods

### Participants and Study Design

This study initially included 24 healthy male and female occasional tobacco users, defined as consuming no more than 10 cigarettes or 10 pouches of Swedish snus per month, aged between 18 and 40 years. Written informed consent and a health declaration were obtained from all participants at the time of inclusion. The study was conducted in accordance with the Declaration of Helsinki (1975) and received approval from the Swedish Ethical Review Authority in Stockholm (No: 2020–03387).

## Study Protocol

A randomized, crossover design was employed. Participants were allocated to initially undergo either Heat-Not-Burn Tobacco Product (HTP) exposure or a control session, when participants breathed ambient air. The sessions were spaced by a minimum one-week washout period. Pre-participation requirements included abstaining from nicotine products, anti-inflammatory medications, and strenuous physical activity for one week. Alcohol and caffeine intake were prohibited 24 h before each session. Exclusion criteria encompassed any chronic disease, recent infections or inflammatory conditions within seven days of participation, pregnancy, and a BMI exceeding 30 kg/m^2^. Sessions commenced after an eight-hour fasting period, with blood sampling performed following 15 min of rest in a semi-supine position within a temperature-controlled environment (21–23 °C).

## Exposure and Blood sampling

Under research assistant supervision, participants were exposed to HTP using an IQOS 3 Multi device (Philip Morris International) for the exposure sessions. Each session consisted of 28 inhalations, one every 30 s, for 14 min, with each inhalation lasting approximately 2–3 s. The tobacco pods (Sienna heats stick, Philip Morris International) contained 203.3 mg tobacco (25.6%), 47.1 mg glycerol (5.9%), 2.8 mg propylene glycol (0.347%), and delivered an estimated 1.4 mg of nicotine per pod.

Blood samples were collected from the antecubital vein at baseline (for routine blood tests and EV analysis) and 4 h post-exposure. Baseline blood tests included a complete blood cell count, electrolytes, serum creatinine, lipid profile, and glucose levels. For extracellular vesicle (EV) analysis, blood samples were collected into citrated tubes and promptly centrifuged at 2000 g for 20 min at room temperature (RT), no more than one-hour post-collection. This process yielded platelet-poor plasma (PPP), which was immediately transferred to storage at − 80 °C to preserve sample integrity for subsequent EV analysis.

## Extracellular Vesicle Measurement

Platelet-poor plasma (PPP) samples were thawed at 37 °C for approximately 5 min before undergoing centrifugation at 2000 g for 20 min at room temperature (RT). The resulting supernatant was further centrifuged at 13,000 g for 2 min. The two centrifugation steps after thawing are performed in order to remove any large debris. A 20 μL sample was placed into a 96-well plate and incubated for 20 min in the dark with 5 µL of lactadherin-FITC (Hematologic Technologies, Essex Junction, VT, USA) and either 5 µL CD41-PE for platelet-derived EVs (PEVs) or 5 µL CD106-APC for endothelial EVs, sourced from Beckman Coulter (Brea, CA, USA). Moreover, Leukocyte-derived EVs as well as Neutrophil-derived EVs were investigated by labelling EVs with lactadherin and 5 µl of CD45 or CD15 respectively (Beckman Coulter (Brea, CA, USA). The samples were then diluted with 120 μL of CytoFLEX Sheath Fluid (Beckman Coulter, Brea, CA, USA) prior to analysis by a CytoFLEX flow cytometer (Beckman Coulter). To establish the gating for EV analysis, Nano fluorescent Yellow Particles of 0.13 μm, 0.22 μm, 0.45 μm, 0.88 μm, and 1.35 μm (Spherotech, Lake Forest, IL, USA) were utilized, detecting a size range of approximately 0.2 to 1.0 μm. The lower EV gate was set at the 0.2 μm beads to avoid the significant background noise present around 0.13 μm, which could affect accurate measurements. Additionally, the gating strategy was verified using labeled and unlabeled EVs, as beads and EVs have different refractive index. Unstained EVs, isotype controls, single fluorochrome-stained EVs, and EVs stained as fluorescence-minus-one (FMO) controls were employed to calibrate the instrument. The instrument’s threshold was set to violet side scatter. EV concentrations are reported in units per µL of plasma, derived from the 20 µL sample prepared for flow cytometry. The intra- and inter-assay variability for the flow cytometry analysis was maintained below 10%.

## Statistical Analysis

Data were analyzed using GraphPad Prism version 8.0 (GraphPad Software Inc., US) and IBM SPSS Statistics version 26 (IBM Corp., US). The normality of the data distribution was assessed through visual inspection and a skewness test. An observed skewness in the data for leukocyte-derived EVs led to the exclusion of one outlier from further analysis. Differences in EV concentrations across time points were evaluated using a two-way repeated measures ANOVA. Statistical significance was determined at a *p*-value of < 0.05. All statistical analyses were conducted by an investigator who was blinded to the group allocations.

## Results

A total of 23 participants were included in the analysis. Consistent with the methodology described one outlier in the control group was excluded from the analysis. Comprehensive demographic and baseline laboratory data for all subjects can be found in Table 1.

**Table 1:** Subject baseline characteristics

	*n* = 23
Male/female	12/11
	mean ± SD
Age [years]	26 ± 5
BMI (body mass index) [kg/m2]	24.2 ± 3.4
Waist circumference [cm]	81 ± 10
Hemoglobin [g/L]	137 ± 11
White blood cells [× 10⁹/L]	5.7 ± 1.9
Platelets [× 10⁹/L]	242 ± 53
Creatinine [µmol/L]	72 ± 13
Glucose [mmol/L]	5.2 ± 0.3
Cholesterol [mmol/L]	4.4 ± 0.9
Low density lipoprotein [mmol/L]	2.4 ± 0.8
Triglycerides [mmol/L]	0.8 ± 0.3
Alanine transaminase [µkat/L]	0.3 ± 0.1

**Fig. 1:**
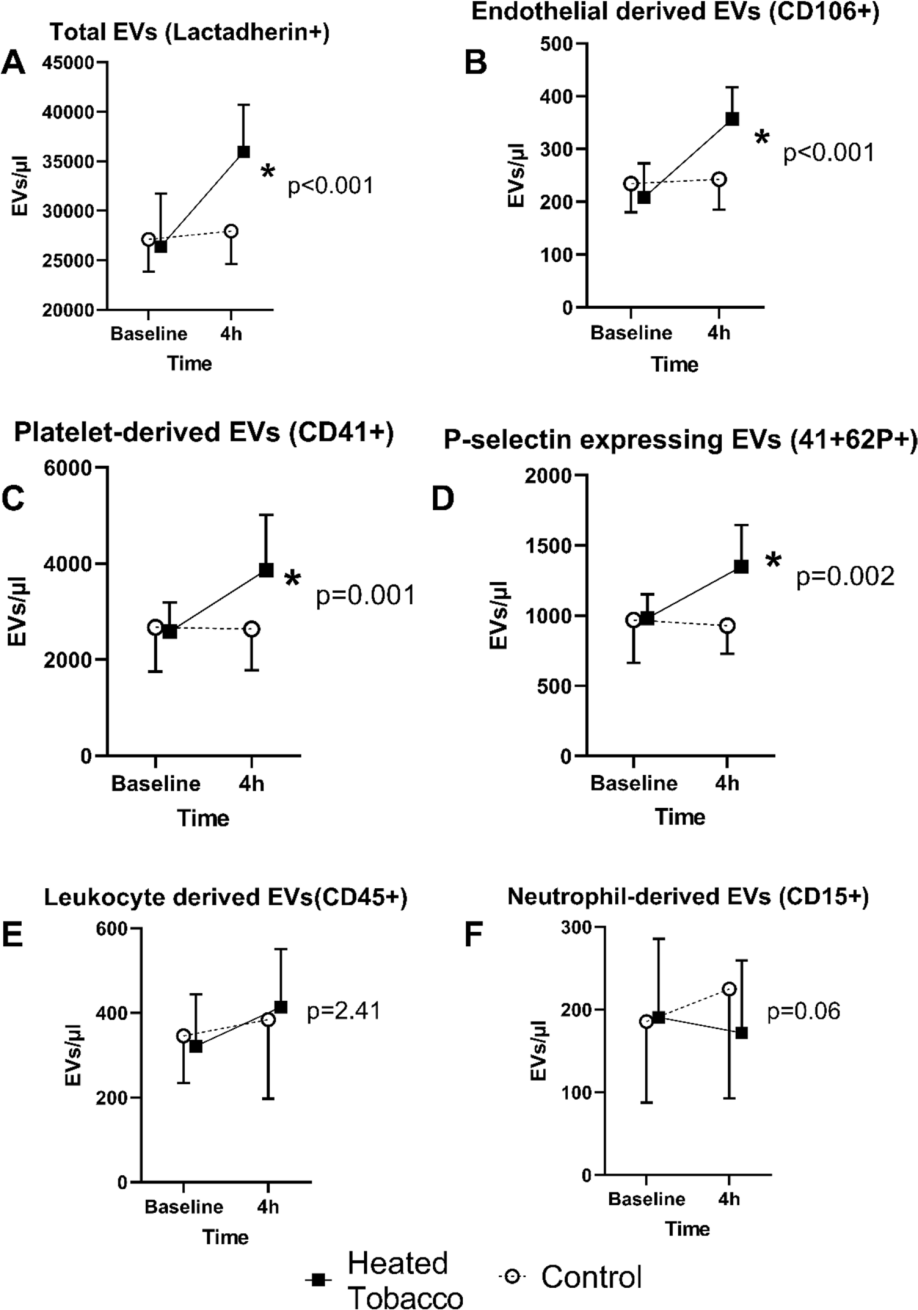
Changes in mean extracellular vesicle (EV) concentrations ± SD, post-exposure to heated tobacco products and control conditions over time. Sub-panels represent different EV populations: **A** Total EVs, **B** EVs of endothelial origin, **C** EVs of platelet origin, **D** EVs from platelets, expressing p-selectin, **E** EVs derived from leukocytes, and **F** EVs derived from neutrophils. Statistical significance of the time*exposure interaction effect was assessed using multiple measures ANOVA, with p-values indicating the outcomes

Extracellular vesicles were characterized by flow cytometry based on protein expression, as outlined in the Methods section. The findings are detailed in Fig. 1 and Online Resource 1. In summary, total EV counts exhibited a significant increase after Heated Tobacco Product (HTP) inhalation. Specifically, EVs of endothelial and platelet origin showed significant elevations. Additionally, EVs expressing p-selectin were also significantly increased. In contrast, leukocyte- and neutrophil-derived EVs did not display a significant change post-HTP inhalation.

## Discussion

In this investigation, we assessed the acute impacts of HTP-inhalation on EV-levels within a cohort of young, healthy individuals. This study demonstrates that brief exposure to nicotine-rich HTPs significantly increases EV levels, particularly those originating from endothelial and platelet cells. Conversely, EVs of leukocyte and neutrophil origin did not show significant changes following HTP exposure.

Endothelial and platelet-derived EVs are known to increase in response to endothelial activation or damage and are involved in processes such as platelet senescence and activation [19]. The presence of these EVs is a recognized marker of acute vascular distress, commonly observed in serious conditions like myocardial infarction and stroke, where elevated EV levels are correlated with poorer clinical outcomes [12–14]. Particularly noteworthy is the increase in P-selectin (CD62P) positive EVs, which indicates not only an increase in platelet shedding but also significant platelet activation [20].

The linkage between habitual cigarette consumption and an escalated cardiovascular disease risk is well-established, spotlighting endothelial dysfunction as a critical precursor to atherosclerotic pathology [21]. Our previous research confirmed that inhaling smoke from just one cigarette can elevate EV levels from endothelial, platelet, and leukocyte origins, accompanied by an increase in circulating endothelial progenitor cells (EPCs). This suggests acute vascular damage, inflammation, and thrombosis [16]. Moreover, in habitual smokers, elevated EVs of endothelial origin are consistently detected and have even been associated with the progression of chronic obstructive pulmonary disease (COPD) and emphysema [22]. Conversely, smoking cessation typically results in a reduction of endothelial EVs, provided there is no permanent pulmonary damage [23]. Acute inhalation of vapor from electronic cigarettes has also been shown to significantly increase endothelial- and platelet-derived EVs, underscoring that non-combustible nicotine delivery products, such as e-cigarettes, can adversely affect vascular health [17, 24]. Collectively, these findings indicate that HTPs elicit effects on EV shedding from endothelial and platelet cells that are comparable to those observed with traditional smoking and e-cigarette use.

Interestingly, our study noted that levels of EVs originating from leukocytes and neutrophils were unaffected. This is in contrast to previous findings where an increase in EVs from leukocytes was observed following cigarette inhalation [16, 25]. Inhalation of e-cigarettes, on the other hand, did not affect leukocyte-derived EVs [24]. The significant amounts of volatile organic compounds present in cigarette smoke could induce strong pro-inflammatory effects, which might explain the differential impact observed in traditional cigarette use compared to other nicotine delivery systems [26].

Historically, adverse vascular outcomes were attributed primarily to the combustible components of cigarettes [27]. However, evidence increasingly highlights a significant role for nicotine in promoting atherosclerotic conditions, challenging the perceived safety of combustion-free alternatives like e-cigarettes and HTPs [28, 29]. For instance, our previous study found that inhaling e-cigarette vapor without nicotine did not increase EV levels, unlike vapor containing nicotine [17]. Nicotine’s influence extends across a spectrum of biological responses. It not only exerts direct sympathomimetic effects but also triggers the release of endogenous catecholamines, which in turn, can activate platelets via adrenergic receptors [30]. These conditions potentially lead to increased production of reactive oxygen species (ROS), exacerbating endothelial inflammatory responses and subsequent EV release [31, 32]. This EV release might serve not merely as a marker of apoptosis but potentially as a protective mechanism, with EVs ferrying antioxidant enzymes to combat ROS [33]. Moreover, studies have shown that EVs isolated from users of e-cigarettes and traditional cigarettes exhibit in vitro vasoconstricting capabilities. This effect is mediated through the diminished activation of endothelial nitric oxide synthase (eNOS) and increased production of endothelin-1 (ET-1), both of which contribute to vascular dysfunction [34].

In a previous analysis of our study participants, published previously, we observed that arterial stiffness increased following inhalation of HTPs [30]. Arterial stiffness serves as a surrogate marker for endothelial function and an independent indicator of vascular health. Increased arterial stiffness is robustly associated with the progression of vascular disease. These findings, coupled with the results from our current EV analysis, strengthen the assertion that HTP inhalation swiftly and significantly impacts vascular function, indicative of impaired endothelial function and pro-thrombotic activity [20, 31]. Given the accumulating evidence highlighting the acute adverse effects of nicotine inhalation, it becomes crucial to explore the long-term impacts of nicotine usage, particularly with the emergence of non-combustible nicotine delivery products.

In earlier research investigating the effects of nicotine on thrombus formation in healthy individuals, we observed that nicotine-containing e-cigarette inhalation led to increased thrombogenicity and impaired vascular function [35]. This growing body of evidence suggests that acute nicotine inhalation has detrimental effects on vascular health. However, the long-term vascular consequences of nicotine use, particularly from non-combustible nicotine delivery systems, remain less well-documented due to their novelty. In animal models, chronic nicotine administration has been shown to induce significant vascular changes and aortic remodeling [31, 36, 37]. In human populations, notably users of Swedish snus (oral moist snuff)—a product historically perceived as less harmful due to its lower content of tobacco nitrosamines—the prolonged use has been linked to increased cardiovascular disease mortality [38, 39]. Our previous findings corroborate this, showing that even apparently healthy chronic snus users exhibit significantly increased arterial stiffness compared to healthy, age-matched non-users [40]. These findings highlight nicotine’s central role in vascular detriment.

Contrary to these findings, nicotine replacement therapy (NRT), often regarded as a safe cessation aid, has not been associated with increased mortality post-myocardial infarction, presenting a stark contrast to the risks related to snus use [38, 41, 42]. The administration route, which influences nicotine bioavailability and serum levels, appears critical in mediating these outcomes. Unlike NRT, which gradually elevates serum nicotine levels, products like HTPs or snus rapidly increase nicotine concentrations [43]. Moreover, nicotine may amplify the adverse effects of other aerosol constituents. For example, e-cigarette inhalation without nicotine led to a significant increase in activated platelet-derived EVs, an effect that was substantially intensified with the addition of nicotine [17].

It is also important to note that HTP aerosols contain other potentially hazardous components, such as nitrosamines, reactive oxygen species, volatile organic compounds, and carbon monoxide, albeit at lower levels compared to conventional cigarettes [3]. These primarily combustion-derived constituents are known to induce vascular stress and endothelial dysfunction [21, 44]. The interaction of these components with nicotine could exacerbate their negative impact on the vascular wall. Proposed mechanisms through which nicotine exacerbates these effects include increased adhesion of pro-inflammatory cells, heightened endothelial cell apoptosis, disruption of intercellular communication, and altered proliferation of vascular smooth muscle cells [29].

## Limitations and Strengths

The study focuses on the acute effects of HTP inhalation, limiting its ability to predict long-term health outcomes or chronic vascular changes resulting from sustained HTP use. Without longitudinal data, it’s challenging to ascertain to which degree the observed increase in EVs translates to an increased risk of cardiovascular disease over time. Furthermore, focusing on young, healthy individuals may not capture the full spectrum of potential effects in a more diverse population, including older adults or individuals with preexisting health conditions. The findings may not be generalizable to all HTP products or usage patterns, given the specific conditions under which the study was conducted (e.g., type of HTP used, duration and frequency of inhalation). The timing for EVs to revert to baseline values remains unclear. Previous analyses from cigarette smoking studies indicate that EV levels peak between 1 to 4 h post-inhalation and return to baseline within 24 h [16]. An additional limitation of this study is the absence of urine sample collection to confirm nicotine abstinence, which could have provided purer observed effects.

This study is among the first to investigate the acute effects of HTP inhalation on EV levels in young, healthy individuals, contributing novel insights into the cardiovascular implications of using such products. The acute exposure model and subsequent immediate measurement of EVs offer a controlled environment to directly assess the impact of HTP inhalation, minimizing confounding variables present in observational studies.

## Conclusion

This study highlights a significant increase in EVs of endothelial and platelet origin, suggesting acute vascular stress following HTP inhalation. These findings extend our understanding of the vascular impact of nicotine delivery systems, emphasizing that even short-term exposure to nicotine-containing HTPs can induce vascular stress markers akin to those observed with traditional cigarette and e-cigarette use. While the study underscores the potential vascular risks associated with HTP inhalation, it also highlights the necessity for further research to explore the long-term health consequences of HTP use. Given the increasing popularity of HTPs, especially among younger populations, these results underscore the urgent need for comprehensive regulatory measures and public health strategies aimed at mitigating the cardiovascular risks associated with nicotine and HTPs.

## Supplementary Information

Below is the link to the electronic supplementary material.

Supplementary file1 (DOCX 21 KB)

Supplementary file2 (XLSX 2746 KB)

## Data Availability

Data is provided as supplementary information file.
