# Supplementary material for: Vascular Stress Markers Following Inhalation of Heated Tobacco Products: A Study on Extracellular Vesicles
Source: Cardiovasc Toxicol. 2024 Oct 29;25(1):1–8. doi: 10.1007/s12012-024-09934-6 (PMC11739193; doi:10.1007/s12012-024-09934-6)
Supplement: Supplementary file 1 — Supplementary file1 (DOCX 21 KB) [file 12012_2024_9934_MOESM1_ESM.docx]

**Online Resources**

**Online Resource 1:** Mean extracellular vesicle (EV) concentrations ± SD, post-exposure to heated tobacco products and control conditions over time (baseline and after 4h). Statistical significance of the time*exposure interaction effect was assessed using multiple measures ANOVA, with *p*-values indicating the outcomes.

|  | HTP n=23 | | Control n=22 | | ANOVA for time*exposure |
| --- | --- | --- | --- | --- | --- |
|  | mean | SD | mean | SD | *p*-value |
| PS positive EVs (Lactadherin) base | 26388 | 5371 | 27105 | 3264 | <0.01 |
| PS positive EVs (Lactadherin) 4h | 35944 | 4772 | 27951 | 3322 |  |
| Platelet-derived (CD41) base | 2584 | 609 | 2673 | 920 | 0.01 |
| Platelet-derived (CD41) 4h | 3867 | 1144 | 2638 | 858 |  |
| Platelet-derived, P-Selectin positive (41+62P) base | 983 | 169 | 969 | 305 | 0.02 |
| Platelet -derived, P-Selectin positive (41+62P) 4h | 1349 | 297 | 929 | 200 |  |
| Endothelial-derived (CD106) base | 208 | 64 | 234 | 54 | <0.01 |
| Endothelial-derived (CD106) 4h | 357 | 60 | 243 | 58 |  |
| Neutrophil-derived (CD15) base | 191 | 95 | 170 | 64 | 0.06 |
| Neutrophil-derived (CD15) 4h | 172 | 88 | 207 | 102 |  |
| Leukocyte-derived (CD45) Baseline | 322 | 122 | 343 | 113 | 2.41 |
| Leukocyte-derived (CD45) 4h | 414 | 136 | 357 | 138 |  |
